# Electrochemical Determination of β-Lactoglobulin Employing a Polystyrene Bead-Modified Carbon Nanotube Ink

**DOI:** 10.3390/bios8040109

**Published:** 2018-11-15

**Authors:** Judith Molinari, Laura Florez, Anahí Medrano, Leandro Monsalve, Gabriel Ybarra

**Affiliations:** 1 U.T. Nanomateriales, INTI-Procesos Superficiales, Instituto Nacional de Tecnología Industrial, Av. Gral. Paz 5445, San Martín B1650WAB, Argentina; molinari@inti.gov.ar (J.M.); lflorez@inti.gov.ar (L.F.); 2 Centro de Micro y Nanoelectrónica, Instituto Nacional de Tecnología Industrial, Av. Gral. Paz 5445, San Martín B1650WAB, Argentina; amedrano@inti.gov.ar (A.M.); monsalve@inti.gov.ar (L.M.); 3 Consejo Nacional de Investigaciones Científicas y Técnicas (CONICET), Buenos Aires C1033AAJ, Argentina

**Keywords:** electrochemical biosensor, carbon nanotubes, food allergen, β-lactoglobulin

## Abstract

In this article, we introduce the use of a carboxy-functionalized waterborne carbon nanotube ink for the fabrication of an amperometric biosensor aimed at the quantification of β-lactoglobulin. Detection of this protein from cow’s milk was performed by a sandwich immunoassay onto printed carbon nanotube electrodes. The electrodes were printed using a carbon nanotube ink modified with polystyrene beads containing a high amount of carboxylic groups for protein immobilization. This strategy showed enhanced sensing performance compared to the use of oxidative treatments for the functionalization of electrodes. These electrodes showed an excellent electrochemical behavior, and proteins could be immobilized on their surface via the carbodiimide reaction. These antibody-immobilized carbon nanotube electrodes allowed for the detection of β-lactoglobulin in sub-ppm concentrations.

## 1. Introduction

Food allergies are a growing worldwide concern due to their impact on food safety and public health. For instance, cow’s milk contains several allergenic proteins, such as casein, β-lactoglobulin, and α-lactalbumin. Any food containing allergens should provide a warning to the consumers about their presence. Several methods for the analysis of food allergens have been developed. Commercially available methods for analyzing allergens in foods are based on mass spectroscopy, polymerase chain reaction (PCR), or immunological techniques [1]. Mass spectroscopy is a technique that requires specific, expensive, state-of-the-art equipment and highly trained personnel, and it is only used in cases where a confirmation is required. PCR is used to detect DNA sequences that codify for allergens. Therefore, it cannot be used to analyze oils, milk, or egg white, where the allergenic proteins must be detected. The immunological techniques available in the market are immunochromatography and ELISA (enzyme-linked immunosorbent assay). Immunochromatography is qualitative and it is used to carry out cleaning checks and rapid screening in production lines. ELISA remains the most used method for the detection and quantification of allergens in foods.

The growing interest of the food industry and the increasing regulations require better, faster, and cheaper quality controls. Furthermore, the need to optimize the production with continuous, on-line analysis has led the research of new analytical methods and devices such as biosensors [2,3]. Electrochemical biosensors are especially attractive because the associated instrumentation to collect and process the signal is affordable and easy to miniaturize. In contrast to the optical detection employed in ELISA, which requires laboratory instrumentation, electrochemical biosensors are easier to miniaturize and integrate into small electronic circuits and portable point on care (POC) equipment. Although a considerable amount of work in the field of biosensors has been carried out in recent years, the development of electrochemical biosensors for the detection of food allergens is quite scarce (see, for instance, reviews given in [4,5,6]). 

The use of nanomaterials provides new strategies for the development of biosensors due to their physical and chemical properties. In particular, electrochemical biosensors with increased selectivity, sensitivity, and reproducibility were obtained when carbon nanotubes (CNTs) were used for the preparation of electrodes [7,8]. CNT-based screen-printed electrodes (SPE) biosensors have been reviewed by Jaiswal et al. [9].

Several challenges concerning the construction of highly sensitive electrochemical biosensors still remain. For instance, the need for protein immobilization, either antigens or antibodies, on the electrode surface requires the presence of functionalized groups (e.g., carboxylic or amino groups) which can act as anchoring points for proteins. The surface functionalization of a carbon electrode often requires the use of an oxidative process such as oxygen plasma [10,11] or oxidative acid treatment, which may impact the conductive and electrochemical behavior of the electrodes [12]. Therefore, there is a considerable interest in the development of electrodes which can be easily prepared, present a good electrochemical response, and can be readily functionalized without the need of further oxidative treatments. 

Another major concern for the development of biosensors for the quantification of food allergens is the need to enhance sensitivity and reach lower limits of detection and quantification. This could be achieved by increasing the surface concentration of immobilized proteins [3].

In this work we present a waterborne CNT ink modified with carboxylated polystyrene beads (PSB) for the preparation of electrodes which were in turn used for the construction of a biosensor for the determination of β-lactoglobulin in final rinse samples of clean-in-place systems of production lines. In particular, they are aimed to be used in the manufacture of hypoallergenic formulas obtained either from milk proteins with different degrees of hydrolysis or from non-dairy proteins such as soy. The modification with PSB follows two objectives. On the one hand, the inclusion of carboxylated PSB could avoid the need for oxidative treatments; thus, the process could be simplified and CNT degradation could be avoided. On the other hand, since the number of carboxylic functionalities increases with the number of PSB used in the preparation of the electrodes, a higher sensitivity could be achieved by increasing the surface concentration of the proteins used for biorecognition. We critically discuss how the inclusion of a functionalized component in a waterborne CNT ink affected both the electrochemical behavior of the electrodes and the performance of the amperometric biosensors fabricated employing the PSB-modified CNT ink for detection of hydrogen peroxide with horseradish peroxidase (HRP) enzymatic electrodes, as well as the detection of β-lactoglobulin with a capture (sandwich) immunosensor.

## 2. Experimental Section

### 2.1. Reagents

N-hydroxysuccinimide (NHS), 1-ethyl-3-(3-dimethylaminopropyl)carbodiimide (EDC), o-phenylenediamine dihydrochloride (OPD), Type I horseradish peroxidase (HRP), β-casein and β-lactoglobulin from bovine milk, and rabbit polyclonal antibody anti-(bovine β-lactoglobulin) were purchased from Sigma-Aldrich (St. Louis, MO, USA), and rabbit polyclonal antibody anti-(bovine β-lactoglobulin)-HRP conjugate was purchased from Abcam (Cambridge, UK). Different buffers were used: phosphate buffer saline (PBS) of pH 7.4 (0.1 M NaH_2_PO_4_ (Mallinckrodt) and 0.15 M NaCl (Biopack)); PBS, 0.5 M NaCl, and 0.2% polysorbate 20 (Biopack); 0.1 M phosphate buffer of pH 7.0 (KH_2_PO_4_ (Merck)); blocking buffer (0.01% polysorbate 20 (Biopack) and 1% gelatin (Merck) in 0.1 M buffer phosphate of pH 7.0); rinsing buffer (0.05% polysorbate 20 (Biopack) in 0.1 M buffer phosphate of pH 7.0); and measurement buffer (PBS 0.1 M KCl, 4 mM hydroquinone, and 1.5 mM hydrogen peroxide (Biopack)).

### 2.2. Optimization of Experimental Parameters and Specificity of the Immunoassay

The concentration of β-lactoglobulin used for the immobilization, the dilutions of the primary and secondary antibodies, the used buffers, the incubation time, and detection parameters were optimized by ELISA based on a protocol developed by Vitkova et al. [13]. The optimized values found for ELISA were tested on the biosensor, and the dilutions of the primary and secondary antibodies were adjusted again to obtain the optimal electrochemical signal.

The polystyrene microplates were filled with 1 and 10 μg mL^−1^ anti-(bovine β-lactoglobulin) rabbit polyclonal antibody in 0.1 M carbonate/bicarbonate buffer of pH 9.0 (50 μL per well) and incubated for 1 h at room temperature. The coated plates were washed three times with 0.5 M PBS buffer containing 0.5 M NaCl and 0.2% polysorbate 20 to remove unbound antigens. Each well was then filled with 50 μL of different concentrations (0, 1, 10, 25, 50, and 100 μg mL^−1^) of β-lactoglobulin from bovine milk in PBS containing 1% gelatin for 2 h at 37 °C and was washed three times with washing buffer, and the mixture was incubated for 1 h at 37 °C 50 μL with 0.1 and 1 μg mL^−1^ anti-(bovine β-lactoglobulin)-HRP conjugated rabbit polyclonal antibody in washing buffer and washed three times with washing buffer. Aliquots (50 μL per well) of 1 mg mL^−1^ OPD with 50 mM citrate/phosphate buffer of pH 5.0 containing 0.3 mg mL^−1^ H_2_O_2_ were incubated during 15 min at room temperature in darkness. The reaction was stopped with 50 μL per well of 2 M H_2_SO_4_. Absorbance was measured at 492 nm. 

The tested values of concentration of β-lactoglobulin used in the immobilization on polystyrene wells were 0, 1, 10, 25, 50, and 100 μg mL^−1^. The used primary antibody dilutions were 1 and 10 μg mL^−1^, while the used dilutions of the secondary antibody were 0.1 and 1 μg mL^−1^. The best results for ELISA were obtained with primary antibody dilutions of 1 and 10 μg mL^−1^ for the immobilization on polystyrene and with dilutions of 0.1 μg mL^−1^ for the secondary antibodies. When the optimized values found for ELISA were used in the electrochemical biosensor, it was found that a higher primary antibody concentration was necessary, so a value of 100 μg mL^−1^ was used.

Since the method proposed in this work is meant to be specific for the detection of β-lactoglobulin in rinse samples obtained after cleaning-in-place of production lines, proteins that could contaminate the surface of the equipment and products were selected and tested for cross reaction. The specificity of the immunoassay was tested against bovine serum albumin, β-casein, and a soy milk extract. Samples of β-casein and bovine serum albumin of different concentrations (0.1, 1, 10 and 100 μg mL^−1^) were prepared in PBS containing 1% gelatin. A soy milk formula (Nutrilon ProExpert, Nutricia Bagó, Batch No. PTL170342/00145 C2, expiration date 5 May 2019) with a weight content of 13% of soy proteins was extracted and diluted according to Ridascreen Fast b-lactoglobulin ELISA kit. It was found that bovine serum albumin and the soy milk extract did not exhibit any significant cross reaction. On the other hand, a cross reactivity of 1% to β-casein was found.

### 2.3. Preparation of the Electrodes

**Figure 1:**
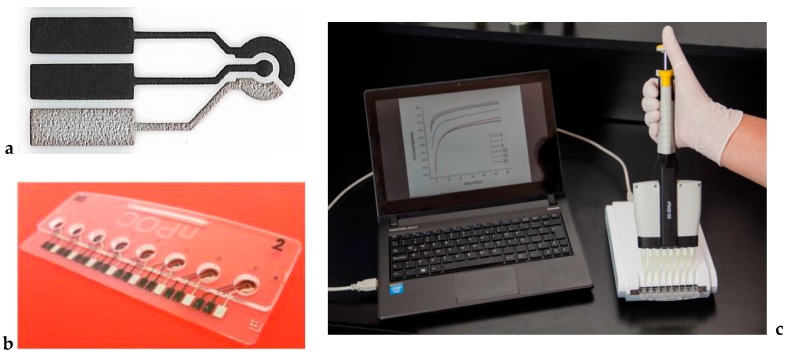
(**a**) Screen-printed electrodes; (**b**) electrochemical cells; (**c**) electrochemical platform comprehending electrochemical cells and electronic instrumentation connected to a laptop via USB port.

Thick film carbon electrodes were printed onto acrylic substrates by screen printing technology as described in [14] (Figure 1a), and the working electrodes were then further coated by drop casting using 0.5 µL with a waterborne CNT ink composed by 2.5% multiwalled carbon nanotubes (Nanocyl, Sambreville, Belgium), acrylic-styrene resin Joncryl^®^ 617, and polyvinylpyrrolidone (PVP K30). This ink, to which 2% carboxylated polystyrene microspheres (Ø 0.9 μm, Sigma Aldrich) was added, was prepared according to our previously described procedure [15]. The electrodes were integrated in an electrochemical cell constructed with poly(methyl-methacrylate) (Figure 1b). The geometrical area of the electrodes was 0.8 mm^2^. Oxygen plasma treatment was used in the preparation of CNT ink enzyme electrodes (OPT-CNT). In this case, the CNT ink electrodes were treated with an oxygen plasma to promote the formation of carboxylic groups. A Diener plasma polymerization equipment was used with an oxygen pressure of 1 mbar, a set temperature of 50 °C, and a treatment time of 15 s.

HRP was immobilized according to a previously described procedure [15]. Rabbit polyclonal antibodies anti-(bovine β-lactoglobulin) were immobilized onto the electrode surface by means of the carbodiimide reaction [16] between the primary amino groups of the antibody and the carboxyl groups located on polystyrene particles immersed in the carbon nanotube ink with 100 μL of 0.1 M EDC and 10 μL of 25 mM NHS for 30 min. After washing, 50 μL of a 100 μg mL^−1^ anti-(bovine β-lactoglobulin) solution in 0.1 M phosphate buffer of pH 7.0 was incubated for 2 h at 37 °C. After washing, the electrodes were incubated overnight at 4 °C in a wet chamber with the blocking buffer. Finally, the electrodes were washed again and ready to be used.

### 2.4. Electrochemical Measurements

Solutions of β-lactoglobulin in PBS of different concentrations ranging from 0.02 to 10 ppm were incubated and added to each electrode for 2 h at 37 °C. Washed electrodes were incubated with 0.1 μg mL^−1^ anti-(bovine β- lactoglobulin)-HRP conjugated antibody in PBS in the same conditions. Electrochemical measurements were carried out at 25 °C in 50 μL of a PBS buffer of pH 7, 0.1 M KCl, 4 mM hydroquinone (redox mediator), and 1.5 mM H_2_O_2_. A portable potentiostat Nanopoc [17] was used for the amperometric measurements (Figure 1c). Potentials were measured and referred to in the text against a Ag|AgCl|0.1 M KCl reference electrode. For the amperometric measurements, the working electrode potential was set at −0.280 V, and the resulting current was recorded at 60 s. This potential value is negative enough to produce the reduction of hydroquinone to 1,4 benzoquinone under diffusion-controlled conditions [18].

## 3. Results

**Figure 2:**
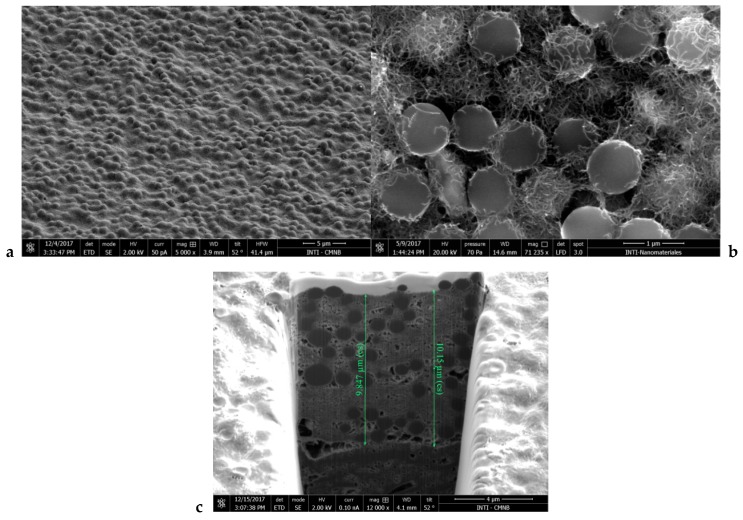
Scanning electron images of electrodes printed with CNT ink with polystyrene beads at magnifications of 5000× (**a**) and 71,235× (**b**), and a cross section obtained by focused ion beam (**c**).

Scanning electron microscopy (SEM) images of electrodes prepared with PSB-modified CNT inks are shown in Figure 2a,b. PSB can be clearly seen in the surface, partially covered by interconnected CNTs. A cross section obtained by a focused ion beam (Figure 2c) showed that PSBs were evenly distributed in the films and that the ink efficiently coated the surface of the SPE, assuring a good electrical contact, with a thickness of around 10 μm.

**Figure 3:**
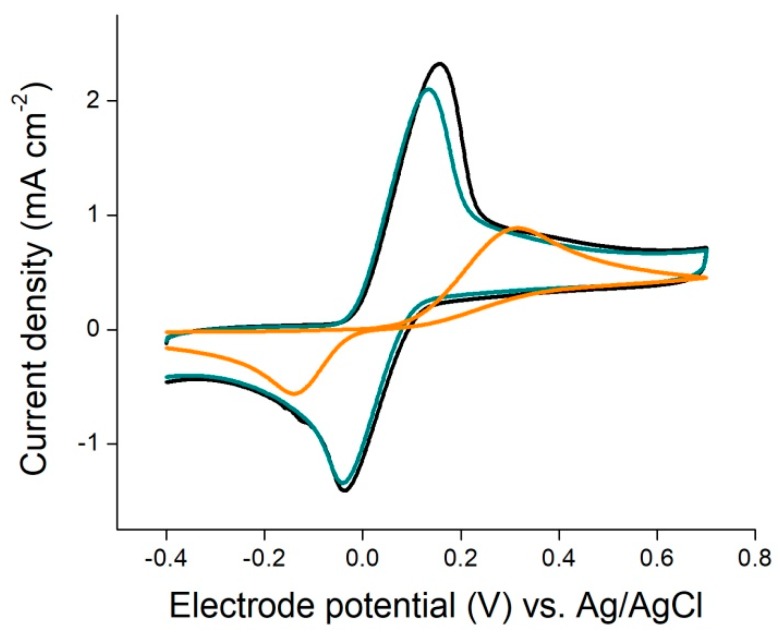
Cyclic voltammograms for hydroquinone at a carbon screen printed electrode (orange), a CNT-ink-coated electrode (black), and a PSB-CNT-coated electrode (green) of 4 mM hydroquinone in a 0.1 M phosphate buffer solution of pH 7.4 at a scan rate of 0.05 V s^−1^.

The electrochemical behavior of the printed electrodes was explored with hydroquinone by cyclic voltammetry. Figure 3 presents the cyclic voltammograms obtained for an uncoated screen printed carbon electrode (SPE), an SPE coated with the CNT ink without PSBs, and an SPE coated with the PSB-modified CNT ink. It can be seen that the cyclic voltammogram obtained for the uncoated SPE presented the biggest peak potential difference, which is associated with quasi-reversible kinetics. When the CNT ink without PSBs was used, the peak potential difference was much smaller and the peak current was significantly higher. This fact has been reported before and is related to the electrocatalytic properties of CNTs toward the oxido-reduction of hydroquinone/bezoquinone [15]. With the inclusion of carboxylated PSB, only a slight decrease in the current was observed, indicating that PSB did not affect significantly the electrochemical behavior of the CNT electrode. 

**Figure 4:**
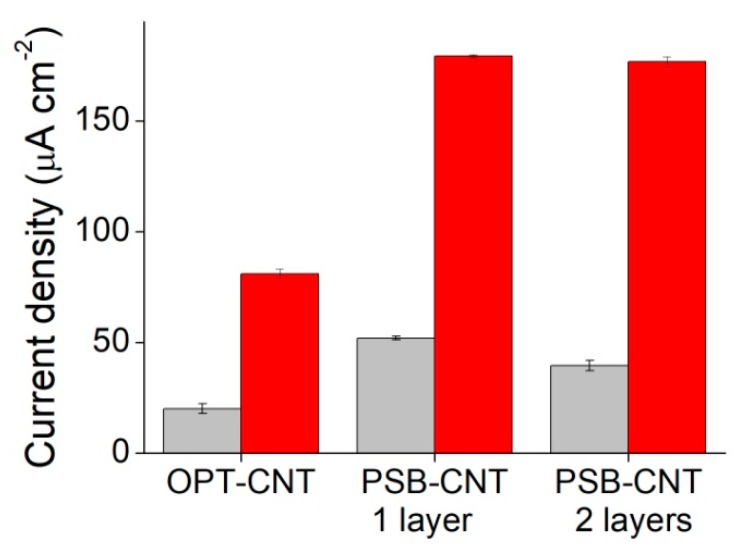
Performance of different HRP enzymatic electrodes. Bars shows the values of current density measured at 60 s at an applied potential of −0.280 V in a PBS buffer containing 4 mM hydroquinone in the absence of H_2_O_2_ (grey) and with the addition of 1.5 mM H_2_O_2_ (red) for oxygen plasma-treated CNT-ink-printed electrodes (OPT-CNT), and carbon SPE coated with one and two layers of PBS-modified CNT ink (PSB-CNT).

In order to assess the performance of PSB-modified CNT ink as a material for the production of enzymatic electrodes, HRP was immobilized onto the electrodes employing the carbodiimide reaction between the carboxylic groups in the beads and the amino groups in the enzyme. These enzymatic electrodes were tested for the quantification of hydrogen peroxide and a linear dependence of the measured current with the concentration of H_2_O_2_ in the 0–1.5 mM range, showing that the immobilized enzymes exhibited a high catalytic activity. These results were compared to HRP enzymatic electrodes prepared with CNT ink electrodes in which oxygen plasma was used for the generation of carboxylic functionalities previous to the immobilization of HRP. An increase in current density of around 100% was found when the PSB-modified CNT ink was used (Figure 4). Enzymatic electrodes were also tested onto SPE with the addition of two layers of CNT ink (20 μm thick) and in this case no further increase in current density was found.

The PSB-modified CNT ink printed electrodes were then used for the production of a portable immunosensor for the quantification of β-lactoglobulin, one of the main milk allergenic proteins. The method was based on a sandwich immunoassay, in which allergens in solution were captured by antibodies immobilized onto the electrode surface and its presence was revealed with HRP-conjugated antibodies. The measured electrochemical signal is thus related to the β-lactoglobulin concentration in the sample solutions, due to the enzymatic activity of HRP-conjugated antibody.

**Figure 5:**
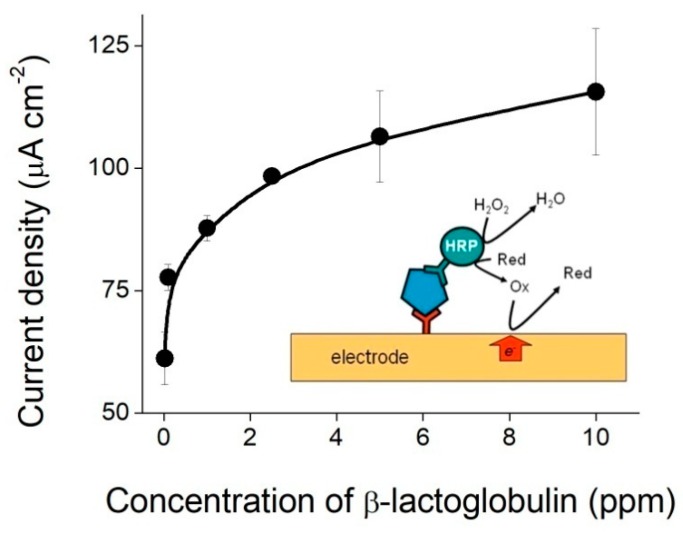
Electrochemical determination of β-lactoglobulin. Dependence of the current measured at 60 s on the β-lactoglobulin concentration at an applied potential of −0.280 V. Error bars were calculated from the standard deviation of three independent experiments. The experimental points were fitted to a power law: *y* = 91.2*x*^0.095^, where *y* corresponds to the current density expressed in μA cm^−2^ and *x* to the concentration of β-lactoglobulin expressed in ppm (*R*^2^ = 0.977). Inset: Schematic representation of the capture immunoassay for the electrochemical determination of β-lactoglobulin (blue pentagon); Red and Ox stand for hydroquinone and 1,4 benzoquinone, respectively.

A schematic representation of the sandwich immunoassay is shown in the inset of Figure 5. After incubation with samples containing different concentrations of β-lactoglobulin and the HRP-conjugated antibody, PBS buffer containing 1.5 mM H_2_O_2_ and 4 mM hydroquinone, which was used as a redox mediator, was placed in contact with the electrodes and current–time curves were collected. The value of the current obtained at 60 s at an applied potential of −0.280 V was found to depend on the concentration of β-lactoglobulin, as shown in Figure 5. The experimental points could be fitted to a power law, and a limit of detection of 0.173 ppm was estimated from the standard deviation of the blank. Using this experimental setup, it was possible to discriminate β-lactoglobulin concentrations ranging from the sub ppm level to 10 ppm. 

## 4. Discussion

As shown in Figure 3, the waterborne CNT ink used in this work presented electrocatalytic properties towards the oxido-reduction of hydroquinone/1,4 benzoquinone, which is often used as a redox mediator in hydrogen peroxide enzymatic biosensors [11]. However, in order to be used in electrochemical biosensors, electrodes must be functionalized to provide anchoring sites for the immobilization of biomolecules. As reported before, oxidative treatments such as chemical oxidation and plasma treatment used for the generation of carboxylic functionalities on the CNT can impair their conductive properties. The introduction of a large number of structural defects may adversely affect their excellent electron transport and mechanical properties [12]. On the other hand, the presence of PSBs on the surface of the electrode might also affect their electrochemical behavior due to partial blocking of their electroactive area. When SPEs coated with the CNT ink without PSBs were used, the peak potential in the voltammograms showed that the oxido-reduction of hydroquinone/benzoquinone was carried out exclusively by CNT, without any sign of the distinctive signal due to underlying carbon SPE. A similar behavior was observed for PSB-CNT-coated SPE electrodes, only affected by a slight decrease in current density, which may be attributed to a slightly smaller CNT active area due to the inclusion of PSBs.

The results obtained with the HRP enzymatic electrodes and the immunosensor in a sandwich configuration confirmed the hypothesis that the inclusion of carboxy-functionalized PSB is a convenient route to avoid the post-functionalization by oxidative treatments. Thus, the preparation of enzymatic electrodes could be simplified without affecting the electrochemical properties of CNTs. Moreover, when compared to oxygen plasma treated CNT ink, an increase in the signal intensity of about 100% was observed with the inclusion of PSB in the CNT ink used for the preparation of enzymatic electrodes. These results suggest that the ink coating with the addition of PSBs allowed an increase in the amount of immobilized HRP and thus a higher sensitivity could be achieved. However, it is apparent that only a fraction of the outmost layer of the ink coating was active for protein immobilization. Otherwise, the current density would have increased linearly with the thickness of the film or the number of layers of PSB-CNT ink. 

Regarding the quantification of β-lactoglobulin, the results obtained using the PSB-modified CNT electrodes show that it is possible to covalently immobilize anti-β-lactoglobulin antibodies with the proper orientation and in sufficient amount as to enhance sensitivity. A limit of detection of approximately 0.2 ppm was estimated. This value is low enough to consider this method as an alternative to those commercially established for milk allergen analysis in clean-in-place practices. A maximum value of 2 ppm of β-lactoglobulin has been proposed as a threshold in final rinse waters in clean-in-place practices [19]. Values for the limit of detection in ELISA typically lie in the 1–5 ppm concentration range [20], and limits of detection lower than 0.2 ppm are unusual in commercially available kits [21]. Recently, electrochemical biosensors developed for food allergen detection with limits of detection ranging from values as low as a few ppbs to ppms have been reported [2,19,20].

In sum, the hypothesis that the combination of a carboxy-functionalized microparticles and CNTs might provide the anchoring sites needed for protein immobilization while preserving mostly unaffected the electrochemical and conductive properties of CNTs has been proved correct. It has also been shown that a higher sensitivity can be achieved in enzyme electrodes by the use of a component (PBS) which increases the amount of immobilized proteins. 

## 5. Conclusions

A waterborne PSB-modified CNT ink was developed and used for printing electrodes which exhibited enhanced electrochemical properties when compared with carbon SPE and plasma treated carbon SPE electrodes. Polystyrene beads containing a high amount of carboxylic groups permitted the direct protein immobilization via the carbodiimide reaction without the need of oxidative treatments and with a negligible adverse impact on the electrochemical performance of carbon nanotubes. Using this modified carbon nanotube ink, a sandwich immunoassay was developed for the determination of β-lactoglobulin, an allergenic protein from cow’s milk, with a detection limit low enough for acceptance as an inexpensive and portable screening test in the food industry.
